# Neuroprotective effect of Apelin 13 on ischemic stroke by activating AMPK/GSK-3β/Nrf2 signaling

**DOI:** 10.1186/s12974-019-1406-7

**Published:** 2019-02-01

**Authors:** Jialin Duan, Jia Cui, Zhifu Yang, Chao Guo, Jinyi Cao, Miaomiao Xi, Yan Weng, Ying Yin, Yanhua Wang, Guo Wei, Boling Qiao, Aidong Wen

**Affiliations:** 1 Department of Pharmacy, Xijing Hospital, Air Force Medical University, No. 127, Changle West Road, Xi’an, 710032 Shaanxi China; 2 Department of Chinese Medicine, School of Life Science, Northwestern University, No. 229, Taibai Road, Xi’an, Shaanxi China

**Keywords:** Ischemic stroke, Apelin 13, Oxidative stress, Inflammation, AMPK/GSK-3β/Nrf2

## Abstract

**Background:**

Previous studies had showed that Apelin 13 could protect against apoptosis induced by ischemic/reperfusion (I/R). However, the mechanisms whereby Apelin 13 protected brain I/R remained to be elucidated. The present study was designed to determine whether Apelin 13 provided protection through AMPK/GSK-3β/Nrf2 pathway.

**Methods:**

In vivo, the I/R model was induced and Apelin 13 was given intracerebroventricularly 15 min before reperfusion. The neurobehavioral scores, infarction volumes, and some cytokines in the brain were measured. For in vitro study, PC12 cells were used. To clarify the mechanisms, proteases inhibitors or siRNA were used. Protein levels were investigated by western blotting.

**Results:**

The results showed that Apelin 13 treatment significantly reduced infarct size, improved neurological outcomes, decreased brain edema, and inhibited cell apoptosis, oxidative stress, and neuroinflammation after I/R. Apelin 13 significantly increased the expression of Nrf2 and the phosphorylation levels of AMPK and GSK-3β. Furthermore, in cultured PC12 cells, the same protective effects were also observed. Silencing Nrf2 gene with its siRNA abolished the Apelin 13’s prevention of I/R-induced PC12 cell injury, oxidative stress, and inflammation. Inhibition of AMPK by its siRNA decreased the level of Apelin 13-induced Nrf2 expression and diminished the protective effects of Apelin 13. The interplay relationship between GSK-3β and Nrf2 was also verified with relative overexpression. Using selective inhibitors, we further identified the upstream of AMPK/GSK-3β/Nrf2 is AR/Gα/PLC/IP3/CaMKK.

**Conclusions:**

In conclusion, the previous results showed that Apelin 13 protected against I/R-induced ROS-mediated inflammation and oxidative stress through activating the AMPK/GSK-3β pathway by AR/Gα/PLC/IP3/CaMKK signaling, and further upregulated the expression of Nrf2-regulated antioxidant enzymes.

## Background

Apelin, a peptide hormone which originally isolated from bovine stomach, is an endogenous ligand of the apelin receptor (AR) [1]. Apelin 13 has the highest activity than other apelin forms [2, 3]. In the central nervous system (CNS), the mRNAs and proteins of AR and apelin are widely distributed in neuronal cell bodies and fibers which suggest that apelin has some pivotal roles in the neuronal signaling pathways [4]. However, the possible protective mechanisms of apelin are largely unknown to date.

Around the world, stroke has been the third leading cause of death and the first leading cause of disability in the adult population [5]. Oxidative stress and post-ischemic inflammatory response are considered to be the key pathogenic mechanisms of the brain injury caused by ischemic stroke [6]. Oxygen and glucose levels rise suddenly during reperfusion, which can potentially aggravate the inflammation, oxidative stress, and cell death already underway due to the initial ischemia [7, 8]. In addition, chronic oxidative stress will lead to the decreased expression of anti-oxidative enzymes and induce the insufficient of antioxidant defense systems, further aggravate inflammation and neuron injuries [9]. Thus, inhibiting the production of ROS or inducing the expression of antioxidant proteins may be useful to inhibit oxidative and inflammation induced by ischemic stroke.

Apelin regulates oxidative stress in various tissues. In myocardial cells, apelin inhibit mitochondrial oxidative damage and lipid peroxidation to protect against oxidative stress and reduce I/R injuries [10]. In kidney tissue, Apelin 13 treatment increases the activity of antioxidant enzymes in a dose-dependent manner and improves renal functions after I/R injury [11]. All the above-mentioned reports strongly suggest that apelin play an antioxidant role in the process of I/R, thus performing its protective effects against I/R injuries in several tissues. However, the possible mechanisms of apelin against oxidative stress and inflammation in brain I/R is understudied.

Among all the antioxidant proteins, nuclear factor erythroid 2-related factor 2 (Nrf2) is very important, which induce more than 500 genes expression involving antioxidant genes and phase II (conjugation) detoxification reactions, and protect the brain from I/R-induced injury [12]. AMP-activated protein kinase (AMPK) is described as the “energy sensor” or “gauge” and express in all cell types. Previous studies reported that AMPK had protective effects against global cerebral ischemia [13], and apelin treatment activated AMPK pathway in brain tissues [14]. Recent researches have showed that the activation of AMPK/Nrf2 pathway protect against ischemic stroke through its anti-inflammatory and anti-oxidative effects [15, 16]. However, the possible mechanism of apelin in activating AMPK and the downstream of them are largely unknown. Considering these points mentioned above, we carried out this study to test the neuroprotective effects of Apelin 13 against the oxidative damage and inflammation and the possible mechanism on focal cerebral I/R injury.

## Methods

### Materials

Apelin 13 peptide was obtained from Santa Cruz Biotechnology (Santa Cruz, CA, USA). Dulbecco’s modified Eagle’s medium (DMEM) was obtained from Hyclone (Logan, UT, USA). Fetal bovine serum (FBS) was obtained from Sijiqing Biotechnology (Hangzhou, China). Annexin V-FITC apoptosis detection kit was obtained from Kaiji Biotechnology (Hangzhou, China). 8-Oxo-dG, MDA, SOD, GSH, CAT, IL-1β, TNF-α, COX-2, and IL-6 detection kits were purchased from Jiancheng Biotechnology (Hangzhou, China). 2,3,5-triphenyltetrazolium chloride (TTC) and dihydroethidium (DHE) were purchased from Sigma-Aldrich Co. (St. Louis, MO, USA). Antibodies for P-AMPK, AMPK, GSK-3β, Nrf2, P-GSK-3β, P-ACC, ACC, NQO-1, HO-1, BCL-2, Bax, cleaved caspase 3, cleaved caspase 9, IP3, PLC, CaMKK, and β-actin were purchased from Cell Signaling Technology (Beverly, MA, USA). Annexin V-FITC apoptosis detection kit was purchased from Jiancheng Biotechnology (Hangzhou, China). All other chemicals used in this experiment were the purest grade commercially available.

### In vivo study

#### Animals and focal cerebral ischemia model

Male Sprague-Dawley rats aged 3 months with the body weight of 200–230 g were purchased from the Experimental Animal Center of the Fourth Military Medical University, and housed in standard cages and controlled environment with 12-h light/dark cycle, 22–25 °C, 45–50% humidity. All of the protocols in this study were approved by the Ethics Committee for Animal Experimentation and performed according to the Guidelines for Animal Experimentation of the Fourth Military Medical University and the National Institute of Health Guide for the Care and Use of Laboratory Animals (NIH Publications No. 80-23) revised in 1996. The preclinical stroke experiments in this study conform to the STAIR-criteria, 5 (randomization, dose-response assessment, blinding, extensive physiological monitoring, and more than one effect measure, [intention to] publish in a peer-review journal) were fulfilled in the current study [17].

Rats were randomly divided into five groups: sham, model, Apelin 13 (30 ug/kg), Apelin 13 (60 ug/kg), Apelin 13 (120 ug/kg). Middle cerebral artery occlusion (MCAO) was induced by intraluminal filament method as previously described [18]. Briefly, rats were anesthetized with chloral hydrate (400 mg/kg, ip), and a 3–0 nylon suture with its tip rounded by heating near a flame advanced from the external carotid artery into the internal carotid artery until it blocked the origin of middle cerebral artery (MCA). Regional cerebral blood flow (rCBF) was measured by transcranial laser Doppler flowmetry (PeriFlux 5000; Perimed AB). A blood-flow drop to 80% of the baseline indicated successful blockage of the middle cerebral artery. After 1.5 h of MCAO, reperfusion was initiated by the withdrawal of the intraluminal suture. Animals were kept in individual cages for 24 h with free access to food and water. Apelin 13 was given intracerebroventricularly 15 min before reperfusion with a stereotaxic frame (Alctt Biotech, Shanghai, China) at a depth of 3.5 mm from the surface of the brain, ± 1.5 mm mediolateral. The doses used in this study were based on our preliminary experiments according to the previous study [19].

#### Infarct volume assessment

Rats were anesthetized with sodium pentobarbital and sacrificed 3 days after MCAO. The brains were harvested after rapid decapitation and cut into slices of 2 mm thickness, and then incubated in a 2% solution of with TTC for 20 min at 37 °C. The volumes for each slice were then summed to determine the whole infarct volume of each brain.

#### Brain water content measurement

The brains were collected from each rats and cut into two sections, the ischemic and non-ischemic. The brain tissues were weighed to get the wet weight (WW) followed dried at 110 °C for 24 h to determine their dry wet (DW). The water content in brain tissues were calculated by using the following formula: (WW − DW)/WW × 100 [20].

#### Neurological deficit evaluation

Neurologic test was determined after reperfusion using modified scoring system developed by Longa [21]. Neurological function was graded on a scale of 0 to 5: 0 = no deficits; 1 = failure to extend left forepaw fully, 2 = circling to the left, 3 = falling to the left, 4 = no spontaneous walking with a depressed level of consciousness, 5 = dead.

#### Determination of malondialdehyde, protein carbonyl, and 8-Oxo-dG levels

MDA, protein carbonyl, and 8-oxo-dG levels in brain tissues were quantified by using commercial kits as stated by the manufacturer.

#### Biochemical analysis

Brain tissues were collected, homogenized in saline, and supernatants were separated. S-100β, NSE, SOD, CAT, GSH, GSH-Px, IL-1, IL-6, and TNF-α levels in brain tissues were measured by commercially available rat ELISA kits as the manufacturer’s protocol.

#### ROS measurement

Six rats from each group were used to measure the levels of ROS in brain tissues by the fluorescence method using 2′, 7′-dichlorodihydrofluorescein diacetate (DCFH-DA). Accumulation of DCF was measured using a spectrofluorometer (Shimadzu Corp., Japan).

### In vitro study

#### Cell culture and I/R model

The neuron-like rat pheochromocytoma cell line PC12 cells is obtained from American Type Culture Collection (Manassas, VA, USA) and cultured in DMEM containing 10% fetal bovine serum and antibiotics (penicillin, 100 IU/ml; streptomycin, 100 μg/ml) at 37 °C in 5% CO_2_ in a humidified incubator. PC12 cells were incubated with DMEM containing apelin or with inhibitors under normoxic conditions for 6 h before I/R.

PC12 cells were pretreated with apelin13 (0.5, 1, and 1.5 μM) for 6 h, and then incubated with Earle’s balanced salt solution (116 mmol/l NaCl, 5.4 mmol/l KCl, 0.8 mmol/l MgSO_4_, l mmol/l NaH_2_PO_4_, 0.9 mmol/l CaCl_2_, and 10 mg/l phenol red) in a hypoxia chamber (Thermo scientific, USA) containing a gas mixture of 95% N_2_ and 5% CO_2_ for 3 h. And then the cells were transferred back to normal culture medium with Apelin 13 under normal culture condition for 6 h to imitate the reperfusion process. The control group was cultured under the normal condition.

#### Analysis of cell viability

Cell viability was studied by using a Cell Counting Kit (CCK8) assay kit (ZETE life Inc., Menlo Park, CA, USA) at 450 nm using a microplate reader (Bio-Rad Laboratory, Hercules, CA, USA). Cell viabilities were presented as values relative to control group.

#### Flow cytometry analysis

Cell apoptosis was measured by an Annexin V-FITC/PI apoptosis detection kit following the manufacturer’s recommended procedure using flow cytometer analysis (BD FACSAria II). A minimum of 10,000 events were read.

#### Determination of malondialdehyde, protein carbonyl, and 8-Oxo-dG levels

MDA, protein carbonyl, and 8-oxo-dG levels in PC12 cells were quantified by using commercial kits as stated by the manufacturer.

#### ROS measurement

ROS levels in PC12 cells were measured by dihydroethidium (DHE) at 5 nM using a laser confocal microscope (Nikon, Japan).

#### Biochemical analysis

PC12 cells were homogenized by RIPA buffer, and proteins were collected. S-100β, NSE, SOD, CAT, GSH, GSH-Px, IL-1, IL-6, and TNF-α levels in PC12 cells were measured by commercially available ELISA kits as the manufacturer’s protocol.

#### siRNA transfection

AMPK and Nrf2-specific short interfering RNA (siRNA) molecules were chemically synthesized by Shanghai Genechem Company. Expression vector pCGN-GSK-3β-Δ9 was provided by Santa Cruz Biotechnology. Transfection was performed by utilizing Lipofectamine RNAiMAX reagent (Life Technologies, CA, USA) as the manufacturer’s protocol. Cells were allowed to recover in fresh growth medium for 48–72 h to permit AMPK and Nrf2 silencing.

### Western blotting

Total protein and nuclear protein extraction was performed after difference treatments in rats’ brain and PC12 cells, and the protein concentrations were measured by BCA Protein Assay reagent kit. Equal amounts of protein samples (30 μg) were separated by 8–12% sodium dodecyl sulfate-polyacrylamide gel electrophoresis (SDS-PAGE) and transferred onto PVDF membranes. The membranes were blocked with 5% nonfat dry milk in TBST for 2 h at room temperature and then incubated overnight at 4 °C with the following primary antibodies: anti-P-AMPK, AMPK, GSK-3β, Nrf2, P-GSK-3β, P-ACC, ACC, Keap1, NQO-1, HO-1, Bcl-2, Bax, cleaved caspase 3, cleaved PARP, cleaved caspase 9, PLC, IP3, CaMKK, Apelin, and β-actin. Then, the membranes were incubated with secondary antibodies (goat anti-rabbit IgG, 1:5000) and visualized by an enhanced chemiluminescent substrate (Thermo, Fisher Scientific). Optical densities of the bands were scanned and quantified image-analysis systems (Bio-Rad, USA). β-actin served as an internal control.

### Statistical analysis

Data from individual experiments were presented as mean ± SD. One-way ANOVA followed by Tukey test was performed by using GraphPad Prism 5.0 (GraphPad Software, La Jolla, CA, USA). *P* < 0.05 was considered to be statistically significant.

## Results

### Apelin 13 protected the brain from MCAO/R-induced injuries

**Fig. 1:**
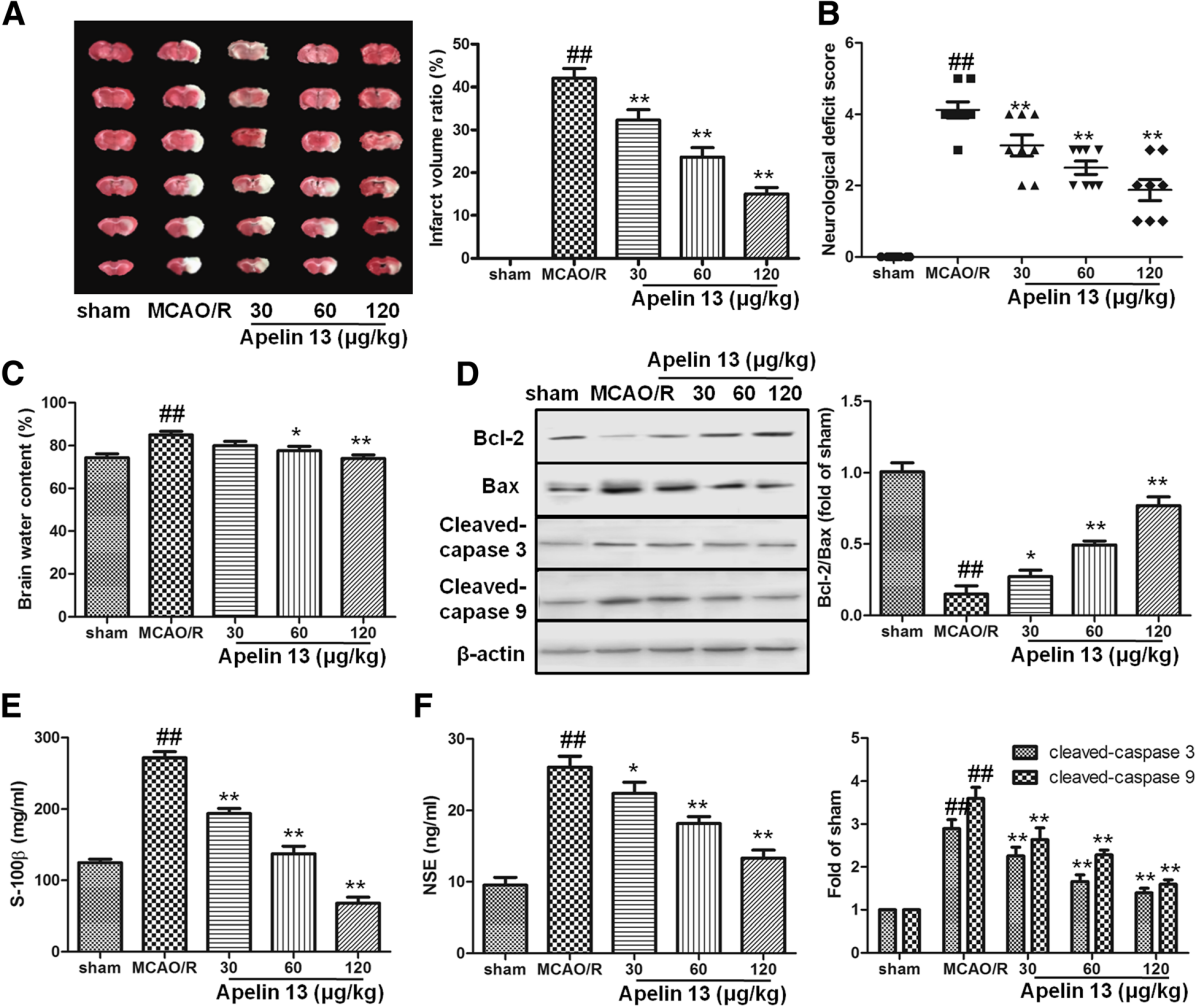
The neuroprotective effects of Apelin 13. **a** Apelin 13 was given intracerebroventricularly 15 min before reperfusion and followed by once daily for 3 days after stroke. TTC staining was performed to evaluate the infarct formation after stroke. **b** Neurological scores in the rats after stroke. **c** Apelin 13 reduced cerebral edema. The water content of ipsilateral hemispheres was measured to evaluate the degree of cerebral edema. **d** Bax, Bcl-2, cleaved caspase 3, and cleaved caspase 9 were detected by western blot as mentioned in the “Methods” section. Levels of S-100β (**e**) and NSE (**f**) in serum were detected by relative kits. ^##^*P* < 0.01 vs. the sham group; *P < 0.05, **P < 0.01 vs. the model group

In sham group, no infarction was observed. MCAO and reperfusion significantly increased the infarct volume ratio in model group (*P* < 0.05), and apelin treatments significantly decreased the infarct volume ratio in a dose-dependent manner (Fig. 1a). Neurological deficit was also observed in rats. Compared with the sham group, rats in MCAO/R group showed strong neurological deficits, suggesting serious neurological deficits were induced by MCAO/R model. Apelin significantly improved the neurological scores compared with the MCAO/R group and ameliorated mental status in rats subjected to MCAO/R (Fig. 1b). Meanwhile, brain edema was estimated by the percentage of brain water content in MCAO/R group, which decreased significantly in apelin treatment groups (60 and 120 μg/kg) (Fig. 1c).

In brain tissues, I/R induces cell apoptosis and further cause more serious injuries in the brain. In this study, western blotting results showed that Bax/Bcl-2, cleaved caspase-3, and cleaved caspase-9 levels were increased significantly in MCAO/R group compared with the sham group (Fig. 1d). Moreover, the presence of apelin significantly decreased these apoptosis markers. S-100β and NSE levels are two brain injury markers. In MCAO/R group, MCAO/R significantly increased the levels of S-100β and NSE, and apelin treatments significantly decreased their levels (Fig. 1e, f). These results suggested that apelin had neuro-protection effects against MCAO/R-induced injuries.

### Apelin 13 protected the brain from MCAO/R-induced oxidative stress and inflammation

**Fig. 2:**
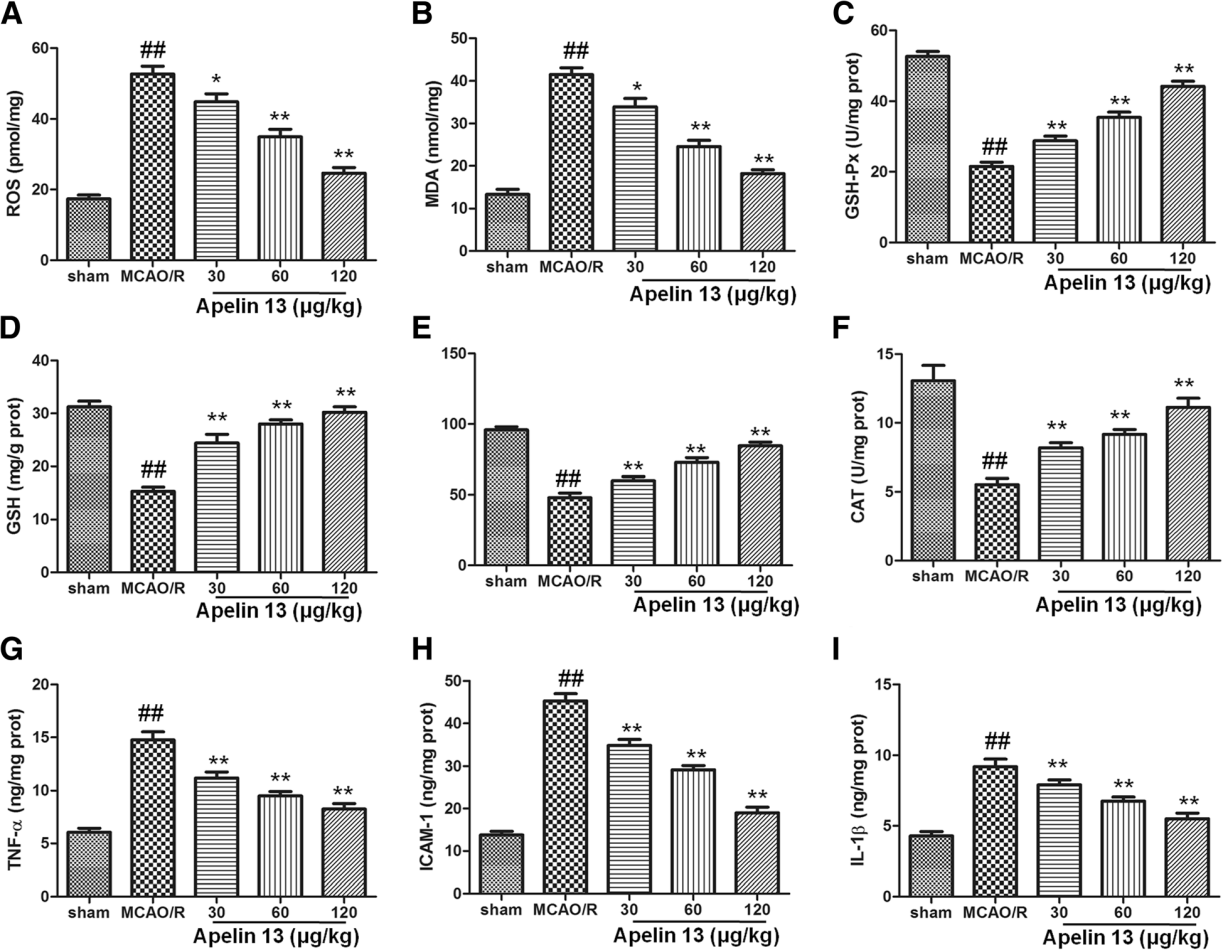
Apelin 13 protected the brain from oxidative stress. The brain tissues were collected after different treatments and homogenized to determine the levels of ROS (**a**), MDA (**b**), GSH-Px (**c**), GSH (**d**), SOD (**e**), CAT (**f**), TNF-α (**g**), ICAM-1 (**h**), and IL-1β (**i**). The protein contents were measured by a BCA protein measurement kit. The results were showed as per milligram proteins or per gram proteins. ^##^*P* < 0.01 vs. the sham group; *P < 0.05, **P < 0.01 vs. the model group

We next examined whether Apelin 13 would be able to induce antioxidant effects in the brain subjected to MCAO/R. As the results in Fig. 2a, b showed, MCAO/R significantly increased the levels of ROS and MDA in MCAO/R group, which compared with the sham group (*P* < 0.01). Treatment with Apelin 13 significantly decreased ROS and MDA levels, and showed a dose dependent manner. Antioxidant proteins are the main substances to eliminate the excess ROS levels in cells, and I/R would decrease their levels. Our results also showed that GSH-Px, GSH, CAT, and SOD levels were significantly decreased in MCAO/R group, compared with the sham group (*P* < 0.01). Apelin 13 significantly increased the antioxidant proteins expression in a dose-dependent manner (Fig. 2c–f).

To assess the anti-inflammatory properties of Apelin 13, TNF-α, ICAM-1, and IL-1α levels in brain tissues are measured. As the results showed in Fig. 2g–i, TNF-α, ICAM-1, and IL-1α levels are increased significantly in rats subjected to MCAO/R. Apelin 13 treatment significantly reduced the levels of TNF-α, ICAM-1, and IL-1α in a dose-dependent manner which compared with that in MCAO/R group (*P* < 0.05). The results suggested that Apelin 13 could protect the brain from MCAO/R-induced oxidative damage and inflammation.

### Apelin 13 activated AMPK/GSK-3β/Nrf2 pathway in rats subjected to MCAO/R

**Fig. 3:**
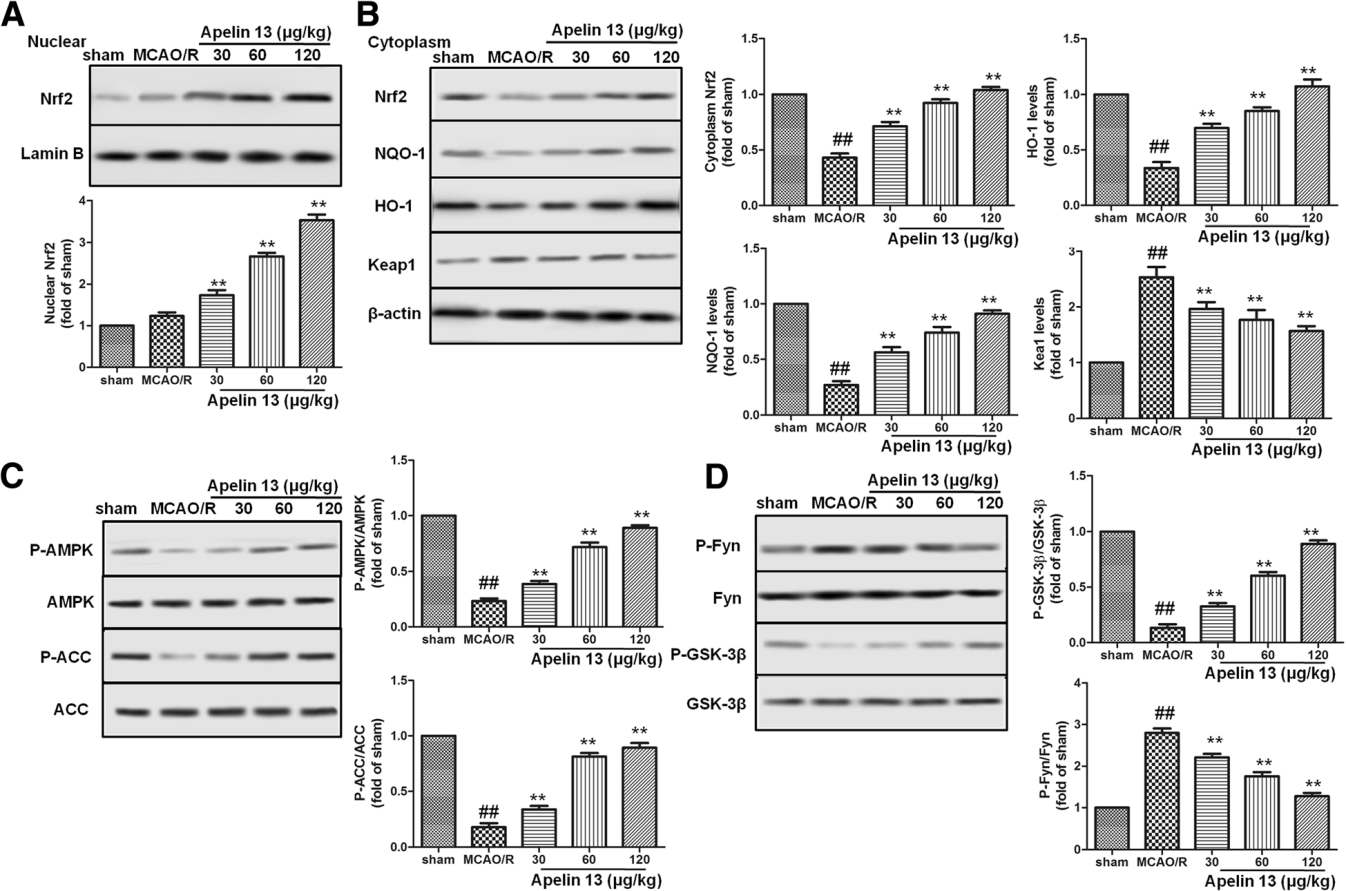
Effects of Apelin 13 on the activation of AMPK/GSK-3β/Nrf2 pathway. **a** Effects of Apelin 13 on the expression levels of Nrf2 in nuclear. Lamin B was used as the internal control protein in nuclear. **b** Effects of Apelin 13 on the expression levels of Nrf2, NQO-1, HO-1, and Keap1 in cytoplasm. β-actin was used as the internal control protein in cytoplasm. **c** Effects of Apelin 13 on the phosphorylation levels of AMPK and ACC. **d** Effects of Apelin 13 on the phosphorylation levels of Fyn and GSK-3β. ^##^*P* < 0.01 vs. the sham group; *P < 0.05, **P < 0.01 vs. the model group

Nrf2 pathway plays very important roles in protecting against oxidative stress-induced injuries in the brain. To further determine the effect of Apelin 13 on the Nrf2 pathway, Nrf2 expression in nuclear and cytoplasm were measured by western blotting. As the results showed in Fig. 3a, Apelin 13 treatment significantly increased the expression levels of Nrf2 in cytoplasm and nuclear compared with MCAO/R group. The downstream proteins of Nrf2, NQO-1, and HO-1 were also increased by Apelin 13 in a dose-dependent manner.

Next, we tried to determine the upstream proteins of Nrf2. The phosphorylation levels of AMPK and GSK-3β were measured by western blotting. As the results showed in Fig. 3c, MCAO/R significantly decreased the phosphorylation levels of AMPK, and its downstream protein ACC compared with sham group. Compared with MCAO/R group, Apelin 13 treatments significantly increased the phosphorylation levels of AMPK and ACC, as well as phosphorylation of GSK-3β at Ser-9. These results suggested that Apelin 13 might protect MCAO/R-induced oxidative stress through AMPK/GSK-3β/Nrf2 pathway in vivo.

### Apelin 13 protected PC12 cells from I/R-induced cytotoxicity

**Fig. 4:**
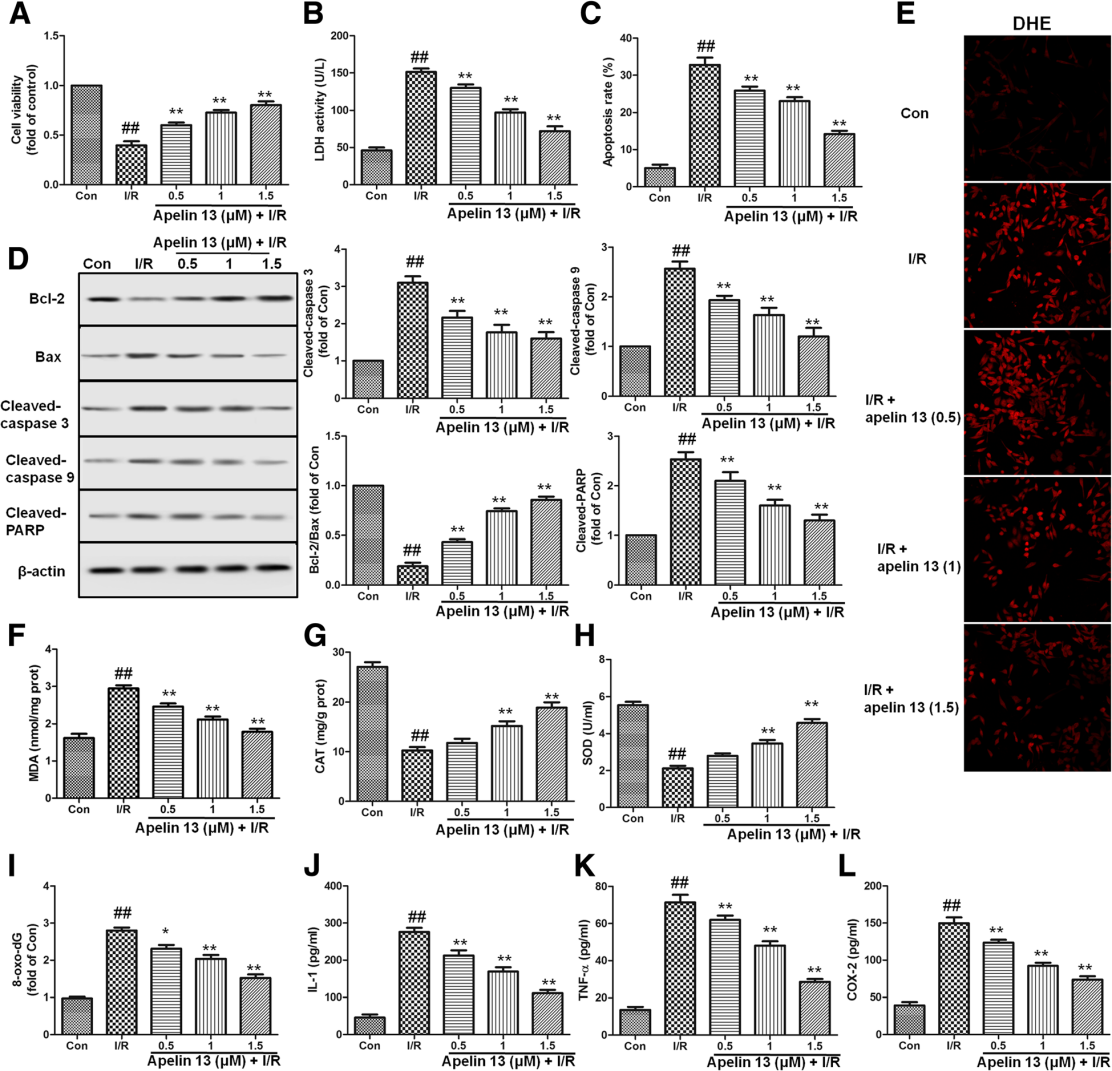
Effects of Apelin 13 on the cytotoxicity and apoptosis induced by I/R in PC12 cells. **a** PC12 cells viability is assessed by measuring the CCK8 reduction. Results were shown as fold of control. **b** Effects of Apelin 13 on the levels of LDH in PC12 cell subjected to I/R. **c** Effects of Apelin 13 on PC12 cells apoptosis induced by I/R. PC12 cells were subjected to I/R with or without Apelin 13 pretreatment, then double stained with Annexin V/propidium iodide (PI). **d** Effects of Apelin 13 on the expression levels of Bax, Bcl-2, cleaved PARP, cleaved caspase-3, and cleaved caspase-9 in PC12 cell subjected to I/R. **e** ROS level is measured by a DHE kit using a laser confocal microscope. The effects of Apelin 13 on the levels of MDA (**f**), CAT (**g**), SOD (**h**), 8-Oxo-dG (**i**), IL-1(**j**), TNF-α(**k**), and COX-2 (**l**) were measured as described in the “Methods” section. The columns and error bars were represented as means ± SD. ^##^*P* < 0.01 vs. the control group; *P < 0.05, **P < 0.01 vs. the I/R treatment group

To estimate the protective effect of Apelin 13 on PC12 cells, cells were pretreated with Apelin 13 (0.5, 1, and 1.5 μM) for 6 h and then subjected to I/R. As the results in Fig. 4a, b showed, I/R decreased cell viability and increased LDH levels significantly; however, Apelin 13 pretreatment significantly deceased cytotoxicity by increasing cell viability and decreasing LDH levels. Apoptosis was determined by flow cytometric. As shown in Fig. 4c, I/R significantly increased the apoptosis rates in PC12 cell compared with control group. Compared with I/R group, Apelin 13 significantly decreased apoptosis rates in a dose-dependent manner. Apelin 13 also significantly suppressed I/R-induced PARP, caspase-3, and caspase-9 cleavage in PC12 cells and increased the ratio of Bcl-2/Bax (Fig. 4d). These results suggested that Apelin 13 may be effective in reversing the cytotoxicity of I/R.

To further investigate the protective effect of Apelin 13, oxidative- and inflammation-related markers are measured. I/R significantly increased the levels of ROS and MDA (Fig. 4e, f), and decreased the levels of SOD, CAT in PC12 cells (*P* < 0.01). Compared with I/R group, Apelin 13 treatment significantly decreased the levels of ROS and MDA, and increased the expression levels of antioxidant proteins (Fig. 4g, h). Oxidative damage in the proteins is measured by the 8-Oxo-dG. 8-Oxo-dG levels were significantly increased in PC12 cells exposed to I/R, and Apelin 13 significantly reduced their levels in a dose-dependent manner (Fig. 4i). The results showed that I/R induced significant increase of IL-1, IL-6, TNF-α, and COX-2 levels in PC12 cells (*P* < 0.01), and Apelin 13 treatment significantly decreased these inflammatory factor levels (Fig. 4j–l). These results suggested that Apelin 13 could protect brain cells from I/R-induced oxidative stress and inflammation in vitro.

### Apelin 13 protected I/R-induced cell injury through Nrf2 pathway

**Fig. 5:**
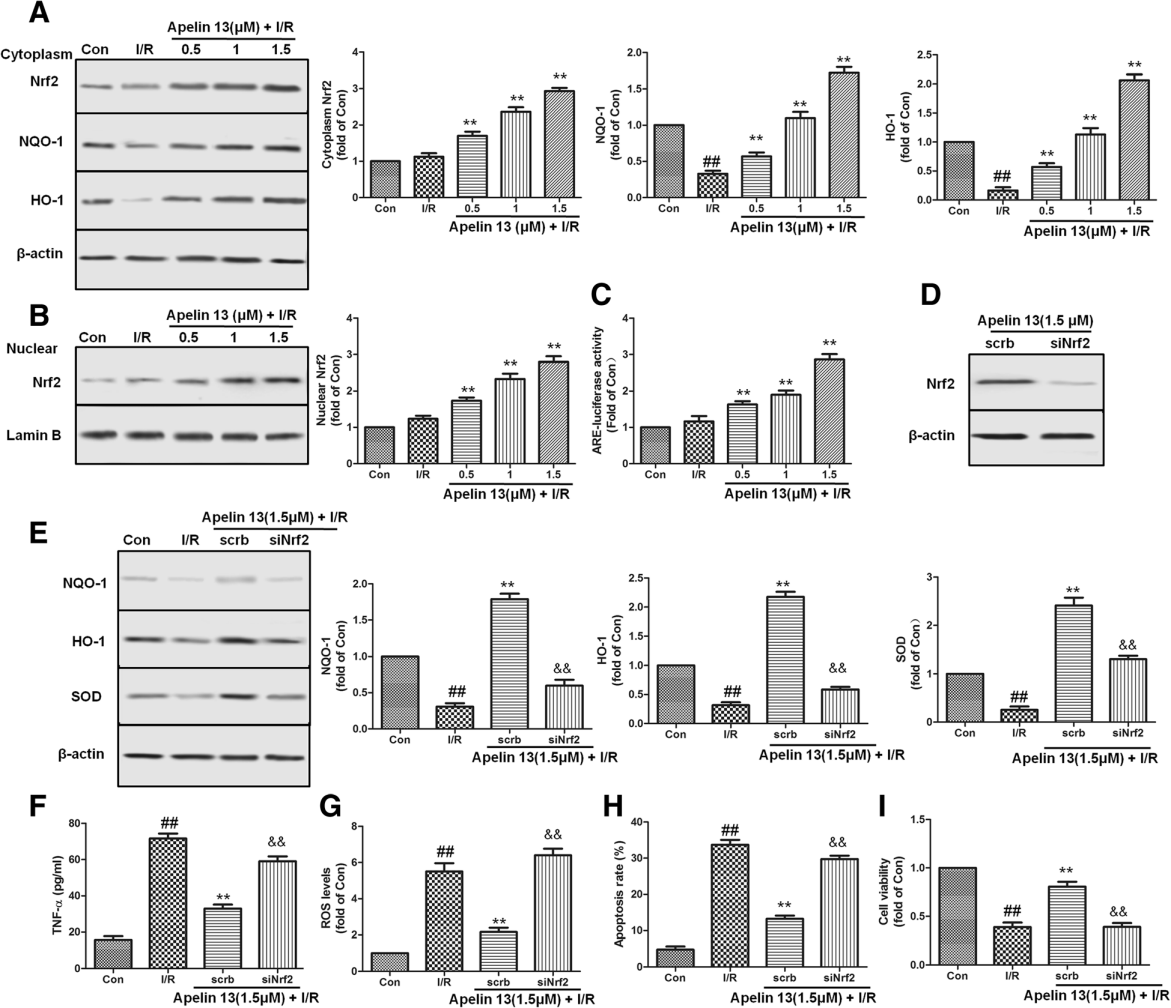
Effects of Apelin 13 on expression of Nrf2-related antioxidant enzymes in PC12 cells subjected to I/R. **a** PC12 cells were pretreated with Apelin 13 for 6 h, then subjected to I/R. The expression levels of Nrf2, NQO-1, and HO-1 in cytoplasm were measured by western bolting as the method motioned in the “Methods” section part. **b** The expression level of Nrf2 in nuclear is measured using the nuclear extract. **c** Effects of Apelin 13 on the activity of ARE determined by ARE luciferase report assay. **d** The cells were treated with Nrf2-specific siRNA (40 nM) or scrb siRNA for 48 h and Nrf2 expression level in cytoplasm was measured by western bolting. **e** Effects of siNrf2 on the protein expression levels of NQO-1, HO-1, and SOD. TNF-α (**f**), ROS (**g**), apoptosis rate (**h**), and cell viability (**i**) were measured after different treatments. The columns and error bars were represented as means ± SD. ^##^*P* < 0.01 vs. the control group; **P < 0.01 vs. the I/R treatment group. ^&&^*P* < 0.05 vs. the scrb control RNA group

To test the mechanism of the apelin, we assessed the expression/activation of Nrf2 pathway in vitro. From western blot analysis, we found that the expression of Nrf2, NQO-1, and HO-1 in Apelin 13 treatment groups were increased significantly compared with the I/R group, and the expression levels of Nrf2 in nuclear were also increased significantly (Fig. 5a, b, *P* < 0.01). In addition, the ARE-luciferase reporter assay results also showed that Apelin 13 increased the transcriptional activity of Nrf2-ARE in a dose-dependent manner (Fig. 5c). Further studies showed that silencing of Nrf2 by using siRNA abrogated the effects of Apelin 13 regarding the modulation of NQO-1, HO-1, and SOD, as depicted in Fig. 5d, e. Nrf2 silencing blocked the effects of Apelin 13 on ROS and TNF-α levels in this experimental model (Fig. 5f, g). Additionally, Nrf2 knockdown suppressed the cytoprotection exerted by Apelin 13 in I/R-treated PC12 cells, which reflected in the changes of cell viability and apoptosis rate (Fig. 5h, i).

### AMPK/GSK-3β contributed to Apelin 13-dependent cell protection effects

**Fig. 6:**
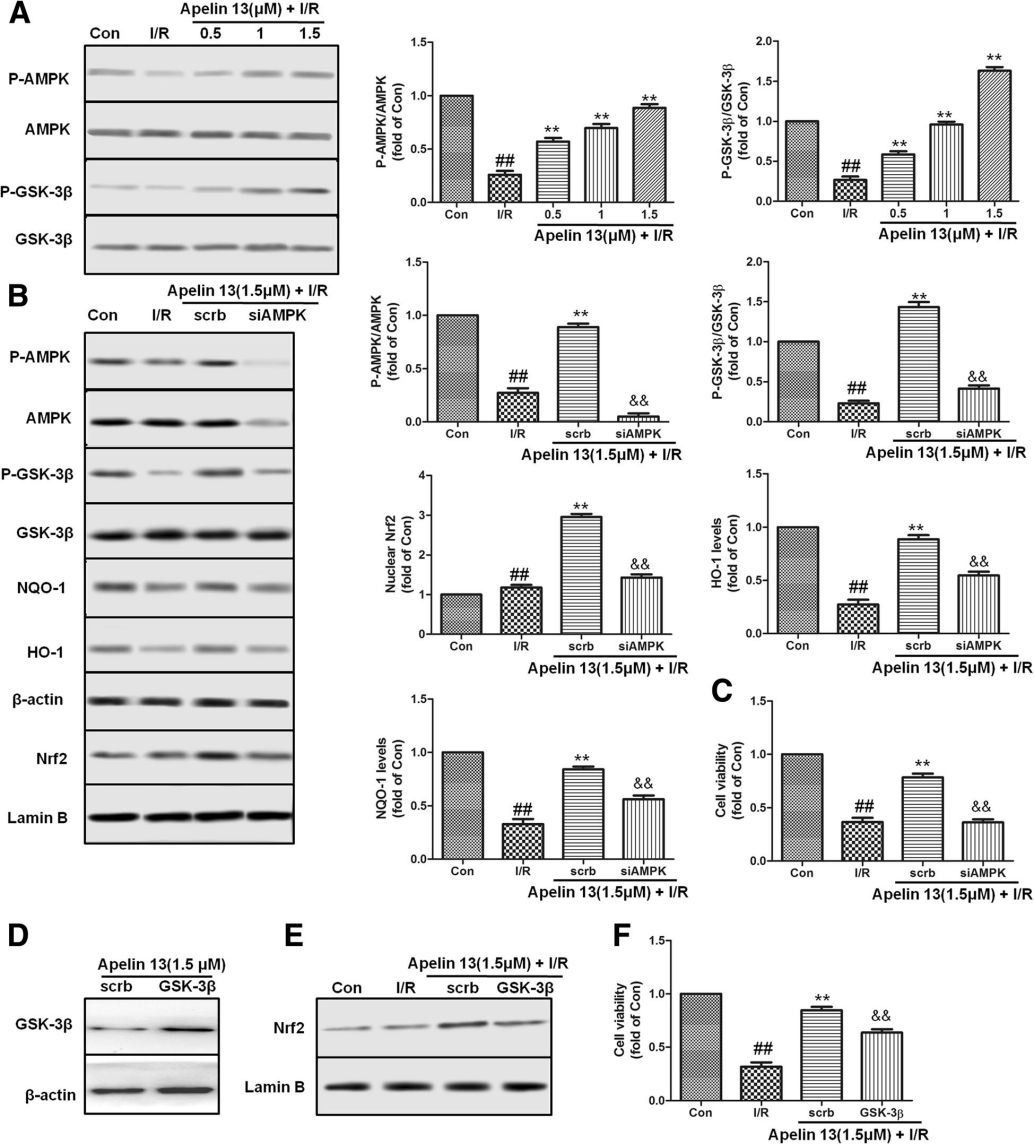
Effects of Apelin 13 were through AMPK and GSK-3β. **a** Apelin 13 increased the phosphorylation levels of AMPK and GSK-3β in a dose-dependent manner. **b** The cells were treated with AMPK-specific siRNA (40 nM) or scrb siRNA for 48 h, then treated with Apelin 13 and I/R, followed by western blotting. **c** Effects of siAMPK on the cell viability. **d** PC12 cells were transfected with the empty vector pcDNA3 or vectors encoding HA-tagged wild-type GSK-3β (WT-GSK3β-HA). Upon transfection, PC12 cells were exposed to different treatments as indicated. **e** Nrf2 nuclear expression levels after transfected with GSK-3β. **f** Effects of GSK-3β-HA on the cell viability. The columns and error bars were represented as means ± SD. ^##^*P* < 0.01 vs. the control group; **P < 0.01 vs. the I/R treatment group. ^&&^*P* < 0.05 vs. the scrb control RNA group

AMPK and GSK-3β plays very important role in protecting brain cell from ischemia-induced injury. To assess the effects of Apelin 13 on AMPK and GSK-3β, the phosphorylation status in PC12 cells were measured. Western blotting results showed that Apelin 13 induced the phosphorylation of AMPK and GSK-3β in a dose-dependent manner which compared with I/R group (Fig. 6a). To study the role of AMPK in cerebral cell protection, cells were transfected with AMPK-specific siRNA. Compared with the scrb control siRNA, AMPK silencing blocked the effects of Apelin 13 on the phosphorylation of GSK-3β (Fig. 6b) and inhibited the expression of Nrf2 and other antioxidant proteins (Fig. 6b). AMPK knockdown also abolished the cytoprotection of Apelin 13 (Fig. 6c). To further study the role of GSK-3β in activating Nrf2 pathway, we over-expressed GSK-3β in PC12 cells. The results showed that the over-expression of GSK-3β attenuated the nuclear translation of Nrf2 and the protective effects by Apelin 13 (Fig. 6d, e). These results indicated that AMPK/GSK-3β/Nrf2 had cascade relationship during Apelin 13 treatment.

### Gα/PLC/IP3/CaMKK was involved in the activation of AMPK pathway

**Fig. 7:**
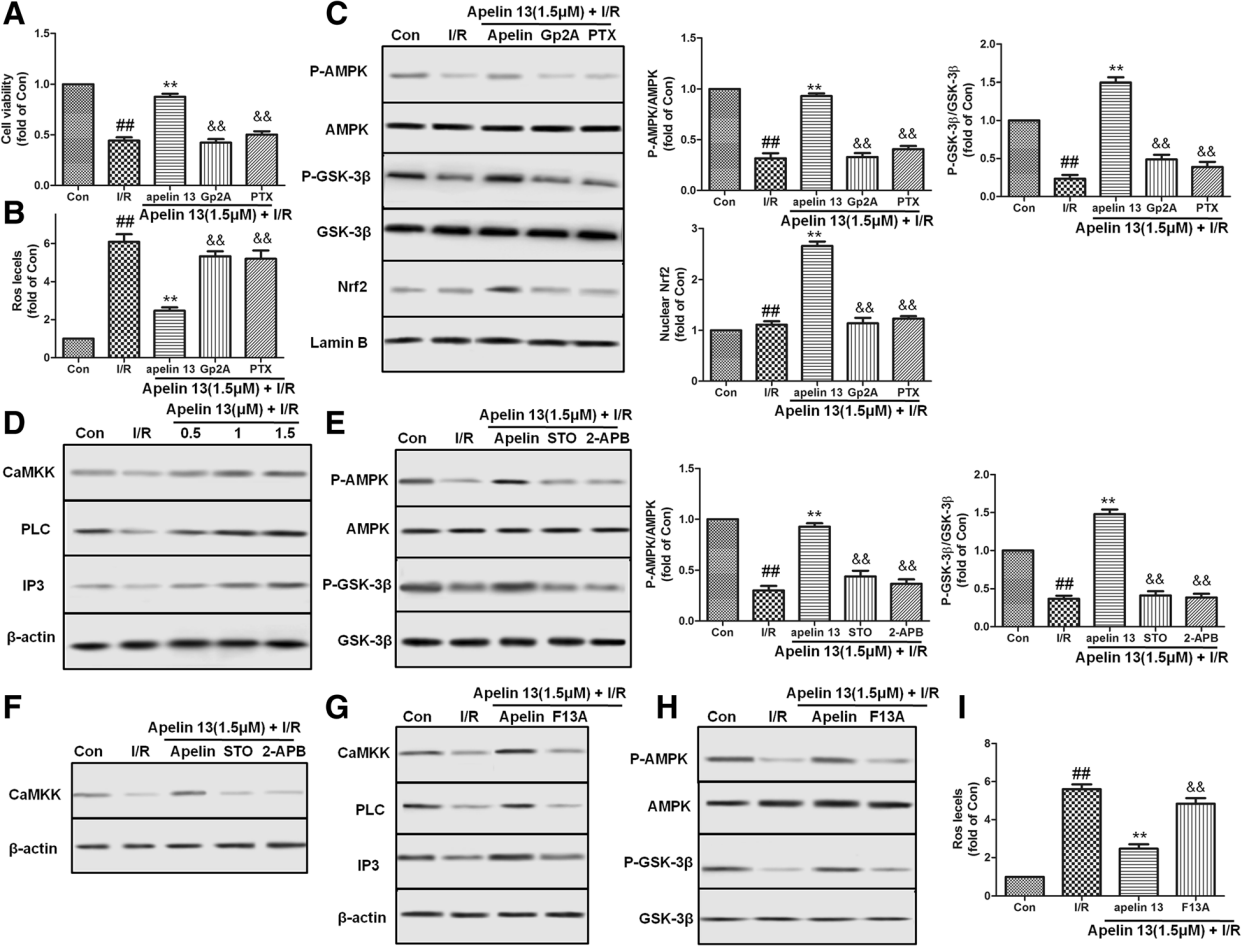
The activation effects of Apelin 13 on AMPK is through AR/Gα/PLC/IP3/CaMKK. PC12 cells were pretreated with Apelin 13 with or without Gα_q_ inhibitor Gp2A (10 μM) and Gα_i_ inhibitor pertussis toxin (PTX) (200 ng/ml), then exposed to the indicated conditions. Cell viability (**a**) and ROS levels (**b**) were measured using relative kit. **c** Gp2A and PTX abolished the effects of Apelin 13 on the activation of AMPK/GSK-3β/Nrf2 pathway. **d** Apelin 13 induced the expression of PLC, IP3, and CaMKK in a dose-dependent manner. **e** PC12 cells were pretreated with Apelin 13 with or without CaMKK inhibitor STO-609 (1 μg/ml) and IP3 inhibitor 2-APB (1 μg/ml), then exposed to the indicated conditions. The phosphorylation of AMPK and GSK-3β (**e**) and the expression of CaMKK (**f**) were determined by western blotting. **g** PC12 cells were pretreated with Apelin 13 with or without apelin receptor inhibitor F13A (1 μM), then exposed to the indicated conditions. F13A inhibited the expression of PLC, IP3, CaMKK (**g**) and the phosphorylation of AMPK and GSK-3β (**h**) and increased the ROS levels (**i**) in PC12 cells. The columns and error bars were represented as means ± SD. ^##^*P* < 0.01 vs. the control group; **P < 0.01 vs. the I/R treatment group. ^&&^*P* < 0.05 vs. the Apelin 13 treatment group

As a kind of GPCR, apelin’s receptor, AR mediates Apelin 13’s effect via Gα subunit, including Gα_i_ and Gα_q_. To determine whether Gα_i_ or Gα_q_ mediates Apelin 13’s effect on I/R-induced PC12 cells injury, Gα_q_ inhibitor Gp2A (10 μM) and Gα_i_ inhibitor pertussis toxin (PTX) (200 ng/ml) were used. The pharmacological inhibition results showed that Gp2A and PTX pretreatment significantly abolished Apelin 13’s cytoprotective effect on I/R-induced PC12 cell injury (Fig. 7a, b). Compared with Apelin 13 treatment, PTX and Gp2A pretreatment significantly reduced the phosphorylation of AMPK and GSK-3β, and inhibited the expression of Nrf2 (Fig. 7c). These data indicated that the rescue effect of Apelin 13 on I/R-induced injury and the activation of AMPK/GSK-3β/Nrf2 pathway were though both Gα_i_ and Gα_q_.

To identify the upstream signal of AMPK activation by Apelin 13 and the relationship between Gα and AMPK pathway, PLC, IP3, and CaMKK expression levels in PC12 cells were measured. After treatment with Apelin 13, the expression of PLC, IP3, and CaMKK were significantly increased compared with the I/R group (Fig. 7c). In contrast, treatment with STO-609 (1 μg/ml), a CaMKK inhibitor, or 2-APB (1 μg/ml), a IP3 inhibitor, both inhibited the phosphorylation of AMPK and GSK-3β by Apelin 13 (Fig. 7d, e). By the way, 2-APB also abolished the expression of CaMKK (Fig. 7f). F13A, an apelin receptor inhibitor, treatment abolished the effects of Apelin 13 on the expression of PLC, IP3, CaMKK, and the phosphorylation of AMPK and GSK-3β (Fig. 7g) and increased ROS levels which inhibited by Apelin 13 treatment. These results showed that AR/Gα/PLC/IP3/CaMKK was involved in the activation of AMPK pathway.

### Apelin 13 regulated AMPK/GSK-3β/Nrf2 pathway through AR/Gα/PLC/IP3/CaMKK in vivo

**Fig. 8:**
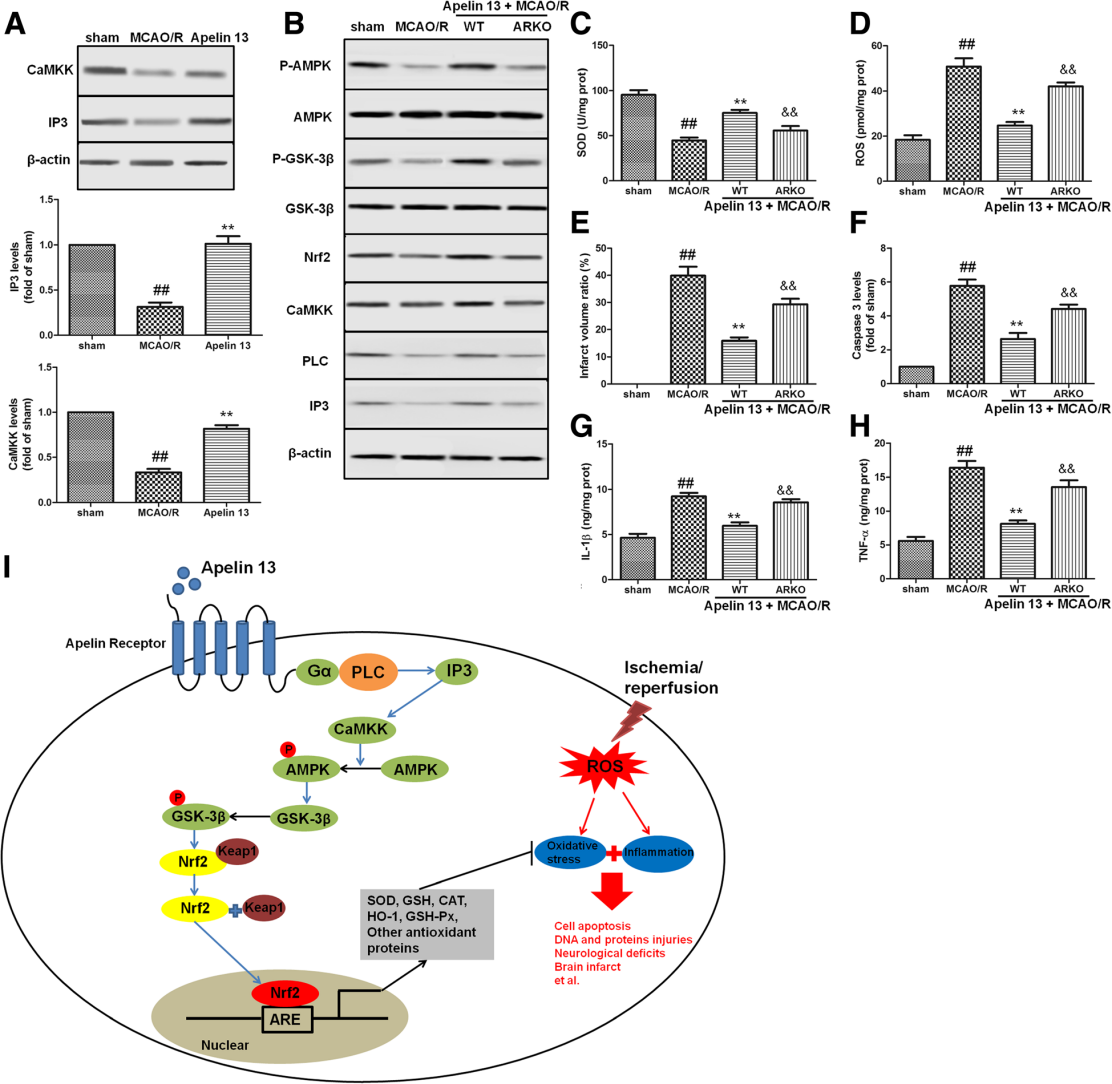
AR/Gα/PLC/IP3/CaMKK is involved in the activation of AMPK/GSK-3β/Nrf2 pathway in vivo*.* **a** The effects of Apelin 13 on the expression of CaMKK and IP3 in the brain. **b** In ARKO rats, the AMPK/GSK-3β/Nrf2 pathway was inhibited and the expression levels of PLC, IP3, CaMKK, and Nrf2 were also decreased. The levels of SOD (**c**), ROS (**d**), infarct volume ratio (**e**), caspase 3 (**f**) IL-1β (**g**), and TNF-α (**h**) were measured as before. The columns and error bars were represented as means ± SD. ^##^*P* < 0.01 vs. the sham group; **P < 0.01 vs. the I/R treatment group. ^&&^*P* < 0.05 vs. the WT rats. **i** Potential mechanism underlying the neuro-protective effects of Apelin 13 on I/R-induced brain injuries in rats and PC12 cells. Apelin 13 protected the brain against ischemic stroke-induced oxidative stress and inflammation through AMPK-mediated inhibitory phosphorylation of GSK-3β downstream of AR/G-coupled receptors pathway, and further induced Nrf2-mediated antioxidant proteins expressions

In order to determine whether the AR/Gα/PLC/IP3/CaMKK was involved in the activation of AMPK/GSK-3β/Nrf2 pathway in vivo, the protein levels of IP3 and CaMKK were determined by immunoblot analysis. We found that MCAO/R resulted in a dramatic decrease in protein levels of IP3 and CaMKK; Apelin 13 treatment significantly increased these proteins expression (Fig. 8a). Compared with wild-type (WT) rats, ARKO rats had decreased phosphorylation levels of AMPK and GSK-3β and reduced expression levels of Nrf2, IP3, PLC, and CaMKK (Fig. 8b). ARKO also decreased the levels of SOD (Fig. 8c) and increased ROS levels (Fig. 8d), infarct volume ratio (Fig. 8e), caspase 3 levels (Fig. 8f), IL-1β levels (Fig. 8g), and TNF-α levels (Fig. 8h), which were inhibited in WT rats treated with Apelin 13.

## Discussion

Around the world, ischemic stroke had been a severe disease and a leading cause of death. Ischemic stroke begins with serious hypoperfusion, which induces excitotoxicity and oxidative damage, and then cause blood-brain barrier dysfunction, microvascular injury, and initiate post-ischemic inflammation [22]. During ischemic stroke, there are two main pathophysiological mechanisms: oxidative stress and inflammation involved. In brain tissue, there are no enough antioxidant defenses to clear the ROS and other free radicals which released by inflammatory cells, so the tissues around the ischemic core are damaged [23]. Many studies had showed that drugs targeting oxidative stress and inflammation are useful in the treatment of I/R-related injuries. In recent years, peptides released from the adipose tissue have been frequently examined in the different tissues I/R studies and indicated that they have significant protective roles [10, 24–26]. As a kind of peptide released from adipose tissue, apelin had showed its protective effects in many physiological processes such energy metabolism and reproduction function [27, 28]. Previous studies also showed that apelin/AR and oxidative stress had strong links in many tissues. Apelin suppresses the levels of ROS in adipocytes, alleviates oxidative stress in cardiomyocytes, and prevents the lipid oxidation in renal I/R [11, 29, 30]. In the nervous system, apelin performs neuroprotective effect against NMDA-mediated excite toxicity and inhibits apoptosis of cortical neurons following brain I/R injury [19, 31]. However, the relationships between apelin, oxidative stress, and inflammation are still largely unknown. Regarding the wide distribution of apelin and its receptor in different regions of the brain, and its excellent activity in other tissues, we hypothesized that the neuroprotective effect of apelin might be dependent on its anti-oxidative and anti-inflammation effects. To clarify these questions, level of oxidative stress and inflammation was measured, and then the mechanisms were studied in in vitro and in vivo I/R model.

In this study, ischemic stroke was induced through MCAO and reperfusion (MCAO/R) operation in rats, and Apelin 13 was given intracerebroventricularly 15 min before reperfusion followed by chronic treatment of daily administration for 3 days. According to the results, Apelin 13 effectively reduced brain infarct size and ameliorated neurological deficits in a dose-dependent manner. We also found that treatment with Apelin 13 significantly attenuated brain edema and levels of brain injury markers (S-100β and NSE) after MCAO/R. During the progression of MCAO/R, neuronal apoptosis, one of the main phenotypes of ischemic cell death, could be induced and even worsen under pathological situations [32]. Bax and Bcl-2 belong to the Bcl-2 family, Bcl-2 is an anti-apoptotic protein, and Bax is a pro-apoptotic protein. Caspases, a family of cysteine proteases, are critical mediators of neuronal apoptosis and neurodegeneration [33]. Cleavage of caspase-3 and cleavage of caspase-9 are activated by I/R and caused DNA fragmentation and mitochondrial dysfunction, thus resulting in the promotion of cell death [34]. The present results indicated that Bax, cleavage of caspase-3, and cleavage of caspase-9 are induced by I/R in rats’ brain and PC12 cells, but Apelin 13 effectively reduced the expression of apoptosis-related protein and induced the expression of Bcl-2. In vitro studies by Annexin-V/PI staining showed that elevation of cell apoptosis ratio by I/R treatment was alleviated by Apelin 13. According to these results, Apelin 13 had protective effects against apoptotic cell death in rats’ brain and PC12 cells.

I/R in the brain induced production of ROS and caused the imbalance between the generation and elimination, thus destroyed the protein, RNA, and DNA and lead to several injuries [35]. In this study, the production of ROS, MDA, TNF-α, ICAM-1, and IL-1β was significantly increased after I/R injury in vitro and in vivo; meanwhile, the levels of GSH-Px, GSH, CAT, and SOD were significantly decreased, indicating that I/R induced oxidative stress and inflammation in brain cells. In Apelin 13 treatment groups, levels of ROS, MDA, TNF-α, ICAM-1, and IL-1β significantly decreased and antioxidant proteins were significantly increased. 8-Oxo-dG levels which are DNA oxidative injury markers are also decreased by Apelin 13 treatment in PC12 cells. These results indicated that the beneficial effects of Apelin 13 might be dependent on its antioxidant and anti-inflammation effects.

Next, we wanted to clarify Apelin 13 through which pathway to induce its effects. Nrf2, a regulator of endogenous antioxidant defense which belonging to CNC-bZIP transcription factors, plays as a chief controller of the detoxification process and intracellular redox status [36, 37]. Under normal conditions, Nrf2 is bound to its chaperone Keap1 and resided in the cytoplasm, and this association promotes its ubiquitination and degradation by some proteasomes [38]. Under oxidative stress conditions, Nrf2 dissociates from Keap1, translocates into the nucleus, binds with antioxidant response elements (AREs), and upregulates transcription of phase II genes, such as glutamate-cysteine ligase, glutathione peroxidases, heme oxygenase-1 (HO-1), and NADPH quinone oxidoreductase (NQO1), that function as antioxidants and anti-inflammatory modulators [39]. Moreover, some reports had showed that Nrf2 plays as an important cytoprotective role in I/R-induced cell injuries, and decreased levels of Nrf2 are found in some neurodegenerative disorders [40]. Therefore, the induction of Nrf2 by some drugs might serve as a strategy not only to promote antioxidant defense, but also to improve neurodegeneration. In this study, compared with the I/R group, Apelin 13-treated brains or cells had a significantly higher level of nuclear Nrf2, together with its downstream NQO-1 and HO-1. Given that Nrf2 nuclear translocation can induce multiple cytoprotective gene expression, Nrf2 nuclear translocation induced by Apelin 13 might mediate the neuroprotective effects we observed in this study. To illustrate this, Nrf2 knockdown experiment was further performed. As the results showed, in Nrf2 knockdown PC12 cells, the antioxidative and cytoprotective effects of Apelin 13 were both suppressed. These results suggested that the protective effects of Apelin 13 in the brain might be through Nrf2 pathway.

Glycogen synthase kinase-3 (GSK-3), a serine/threonine protein kinase constituted by two isoforms, GSK-3α and GSK-3β, participates in several cellular processes including cell apoptosis, oxidative stress, cell proliferation, and glycogen metabolism [41]. Recent evidences had showed that GSK-3β promotes cell death, and inhibition of GSK-3β has been associated with survival mechanism against various stresses that go together with oxidative stress [42]. GSK-3β is inactivated by phosphorylation of serine, and its activity is increased by phosphorylation of tyrosine. In mammals, immunocytochemistry and other analyses results showed that GSK-3β inhibited Nrf2 effects through retaining it in the cytoplasm or increasing Nrf2 export [43]. The relevance of GSK-3β in regulation of Nrf2 had been demonstrated in mice, and long-term oxidative stress leads to GSK-3β activation, downregulated Nrf2, and downstream genes, therefore, limiting the antioxidant cell capacity and increasing the levels of carbonylated proteins and lipid peroxides [44]. In this study, we found that Apelin 13 treatments induced phosphorylation of GSK-3β at serine 9 in a dose- and time-dependent manner. Conversely, Nrf2 nuclear translation and the protective effects by Apelin 13 were attenuated by over-expression of active GSK-3β. These results indicated that the protection of the brain by Apelin 13 might be associated at least in part with GSK-3β phosphorylation.

AMPK is a critical cellular sensor energy balance and expressed widely in neurons throughout the developing of the brain. AMPK can be activated rapidly when cells are subjected to energy-deprived states, for example, ischemia [45]. In hippocampal neurons and SH-SY5Y cells, inhibition of AMPK by compound C inhibited the protective effects of AMPK [13]. Previous studies had showed that the activation of AMPK is responsible for protecting cells from oxidative stress and apoptosis [46]. In hepatocytes, AMPK inhibits GSK-3β activity through Ser9 phosphorylation [47]. Recent study showed Apelin 13 induced AMPK phosphorylation and protected cell apoptosis in cerebral ischemia insults [14]. However, the downstream of AMPK during Apelin 13 treatment is not fully clear. Our results were consistent with the previous study that Apelin 13 induced the phosphorylation of AMPK in dose- and time-dependent manners. AMPK inhibition with siRNA resulted in drastic reduction of phospho-Ser9-GSK-3β levels, and Nrf2 expression also abolished the protective effects of Apelin 13, suggesting that AMPK-mediated phosphorylation of GSK-3β was a plausible mechanism of Apelin 13 in inhibiting GSK-3β activity and inducing Nrf2 accumulation in the nucleus.

Next, we wanted to clarify how Apelin 13 activated the AMPK/GSK-3β/Nrf2 pathway. Angiotensin receptor-like 1 (AR), a kind of G-protein-coupled receptor (GPCR), is an endogenous ligand for apelin. It had been well documented that G-proteins Gα_i_ and Gα_q_ mediated the effects of Apelin 13 and activated different signaling pathways [48]. In rat parotid acinar cells, stimulation of Gα_q_ by carbachol is shown to activate AMPK [49]. Gα_i_ and Gα_q_ were reported to activate the PI3K/AKT and PKC pathways, respectively [50]. Thus, we further determined that Apelin 13’s effect on oxidative stress and AMPK/GSK-3β/Nrf2 pathway was mediated by Gαi or Gαq. The pharmacological inhibition results showed that Gα_i_ and Gα_q_ were both involved in the activation of AMPK/GSK-3β/Nrf2 pathway and protective effects of Apelin 13 against I/R-induced injuries.

Gα_q_ traditionally involved calcium to elicit its effects to activate the AMPK pathway. Among the upstream of AMPK, CaMKK is a calcium/calmodulin-dependent kinase regulation protein kinase which required binding of calcium/calmodulin for their activation [51]. In addition, studies with CaMKK inhibitor, STO-609, showed that effects of Gq-coupled on AMPK were inhibited by CaMKK inhibition. Some reaches also showed that the regulation effect of AMPK activity by Gs- and Gi-coupled receptors is through a CAMKK-dependent pathway [52]. Present results showed that Apelin 13 treatments induced expression of PLC, IP3, and CaMKK protein. Further using pharmacological inhibitors, we demonstrated that Gα, PLC, IP3, and CaMKK were required for Apelin 13-mediated phosphorylation of AMPK and GSK-3β and cytoprotective effects. Compared with wild-type (WT) rats, AR-KO lead to decreased expression of Nrf2, IP3, and CaMKK and increased cell apoptosis and oxidative stress. These findings provided the evidence that Apelin 13 might prevent cell apoptosis after brain I/R injury through AR/G-coupled receptors-mediated AMPK/GSK-3β/Nrf2 pathway in vitro and in vivo experiments.

## Conclusions

Collectively, we demonstrated that Apelin 13 protected the brain and PC12 cells from I/R-induced injuries in vitro and in vivo. These observations were found to be mediated by AMPK-mediated inhibitory phosphorylation of GSK-3β downstream of AR/G-coupled receptors pathway, and further induced Nrf2-mediated antioxidant protein expressions. Thus, Apelin 13 had the potential to pharmacologically defend against I/R-induced oxidative stress and inflammation (Fig. 8i). These data provided new insight into mechanisms by which Apelin 13 and AMPK/GSK-3β/Nrf2 function in cerebral I/R injuries.

## Abbreviations

AMPK: Adenosine 5′-monophosphate (AMP)-activated protein kinase
AR: Apelin receptor
COX-2: Cyclooxygenase-2
DHE: Dihydroethidium
DMEM: Dulbecco’s modified Eagle’s medium
FBS: Fetal bovine serum
GSH: Glutathione
GSK-3β: Glycogen synthase kinase 3 beta
HO-1: Heme oxygenase
I/R: Ischemic/reperfusion
IL-1β: Interleukin-1β
IL-6: Interleukin-6
IP3: Inositol triphosphate
MDA: Malondialdehyde
Nrf2: Nuclear factor (erythroid-derived 2)-like 2
RISK: Reperfusion injury salvage kinase
ROS: Reactive oxygen species
SOD: Superoxide dismutase
TNF-α: Tumor necrosis factor-α
TTC: 2,3,5-triphenyltetrazolium chloride
